# Endothelial cyclooxygenase-1 paradoxically drives local vasoconstriction and atherogenesis despite underpinning prostacyclin generation

**DOI:** 10.1126/sciadv.abf6054

**Published:** 2021-03-19

**Authors:** Jane A. Mitchell, Fisnik Shala, Maria Elisa Lopes Pires, Rachel Y. Loy, Andrew Ravendren, Joshua Benson, Paula Urquhart, Anna Nicolaou, Harvey R. Herschman, Nicholas S. Kirkby

**Affiliations:** 1 Cardio-Respiratory Interface Section, National Heart and Lung Institute, Imperial College London, London, UK.; 2 Laboratory for Lipidomics and Lipid Biology, Division of Pharmacy and Optometry, School of Health Sciences, Faculty of Biology, Medicine and Health, University of Manchester, Manchester Academic Health Science Centre, Manchester M13 9PL, UK.; 3 Department of Molecular and Medical Pharmacology, University of California Los Angeles, Los Angeles, CA, USA.

## Abstract

Endothelial cyclooxygenase-1–derived prostanoids, including prostacyclin, have clear cardioprotective roles associated with their anti-thrombotic potential but have also been suggested to have paradoxical pathological activities within arteries. To date it has not been possible to test the importance of this because no models have been available that separate vascular cyclooxygenase-1 products from those generated elsewhere. Here, we have used unique endothelial-specific cyclooxygenase-1 knockout mice to show that endothelial cyclooxygenase-1 produces both protective and pathological products. Functionally, however, the overall effect of these was to drive pathological responses in the context of both vasoconstriction in vitro and the development of atherosclerosis and vascular inflammation in vivo. These data provide the first demonstration of a pathological role for the vascular cyclooxygenase-1 pathway, highlighting its potential as a therapeutic target. They also emphasize that, across biology, the role of prostanoids is not always predictable due to unique balances of context, products, and receptors.

## Teaser

A paradoxical role for vascular COX-1 products in vasoconstriction and atherogenesis highlights it as a novel therapeutic target.

## INTRODUCTION

Cyclooxygenase enzymes form part of a metabolic cascade that converts arachidonic acid into a range of prostanoid lipid mediators depending on cell-specific expression of downstream synthases. Cyclooxygenase exists as two isoforms: Cyclooxygenase-1 is constitutively expressed in the vascular endothelium, in platelets, and in many other cells in the body. In contrast, cyclooxygenase-2 is constitutively expressed only in limited regions such as the gut and kidney but can be induced elsewhere during inflammation. The prostanoid pathway plays a fundamental role in cardiovascular health (*1*). Much of what we understand of this physiology is based on a simple paradigm of two products acting in balance to control cardiovascular risk: prostacyclin and thromboxane. Prostacyclin is a powerful inhibitor of platelet activation produced by blood vessels, acting via the I-prostanoid (IP) receptor. In isolated large arteries and in plasma, the overwhelming majority of prostacyclin is produced through cyclooxygenase-1 in the endothelium (*1*–*5*). The anti-thrombotic importance of cyclooxygenase-1–derived prostacyclin can be seen in endothelial cell–specific knockout mice, where loss of prostacyclin production is associated with an increase in thrombosis similar to that produced by blockade of the IP receptor (*5*). Cyclooxygenase-2 also protects against thrombosis; its inhibition with nonsteroidal anti-inflammatory drugs is associated with increased risk of cardiovascular events. This anti-thrombotic action is associated with cyclooxygenase-2 expression in discrete tissue locations (*3*) and/or vascular beds (*5*, *6*), but is independent of systemic vascular prostacyclin production (*2*, *5*). The anti-thrombotic activities of vascular prostacyclin are in direct opposition to those of pro-thrombotic thromboxane. Thromboxane is produced by platelets, also through cyclooxygenase-1 (*7*), and acts to drive their activation through binding to T-prostanoid (TP) receptors. This pathway is the target of aspirin, which, at low doses, acts as a platelet-selective cyclooxygenase-1 inhibitor and has proven efficacy in the secondary prevention of cardiovascular events associated with its antiplatelet activity (*8*). Logically, because cyclooxygenase-1 is also expressed in endothelial cells, where it generates anti-thrombotic prostacyclin, anti–cyclooxygenase-1 therapy with low-dose aspirin has been targeted to platelets to maximize benefit by removing platelet-derived thromboxane while preserving vascular cyclooxygenase activity.

A similar protective role for the actions of endothelial prostanoids on the vascular wall is often assumed. Prostacyclin was discovered as prostaglandin X (PGX), a substance released from endothelial cells that could relax rabbit mesenteric and coeliac arteries (*9*), leading to the suggestion that endothelium-derived prostacyclin provides vasodilator tone. Certainly, it is true that exogenous prostacyclin can (i) relax some blood vessels [including mouse mesenteric (*4*, *10*) and renal arteries (*11*)], (ii) increase blood flow in some vascular beds (*12*), (iii) reduce blood pressure (*13*), and (iv) treat pulmonary hypertension (*1*). Even at its discovery, however, it was noted that the vasodilator activity of prostacyclin was vessel specific, having no effect on rabbit aorta, pulmonary artery, or vena cava (*9*), and this was quickly followed by observations that exogenous prostacyclin can act as a vasoconstrictor of pig coronary arteries (*14*) and rat aorta (*15*). It is now clear that this phenomenon of prostacyclin-induced vasoconstriction can be widely demonstrated including in mouse (*4*, *10*, *16*) and human arteries (*17*). Further, endogenous vascular cyclooxygenase pathways, which produce an array of vasoactive prostanoid mediators in addition to prostacyclin, typically provide vasoconstrictor rather than vasodilator tone. For example, cyclooxygenase-1 deletion/inhibition reduces constrictor responses in many isolated vessels including mouse aorta (*4*, *10*, *18*–*20*), renal artery (*11*), carotid artery (*20*), femoral and mesenteric arteries (*4*) and human saphenous vein (*21*), omental arteries (*22*), and umbilical arteries (*23*). This complexity has also been observed in local vascular beds in vivo. In human subjects with or without cardiovascular disease, cyclooxygenase inhibition either reduces or has no effect on basal forearm blood flow but increases blood flow after endothelial activation with acetylcholine (*24*, *25*), consistent with a predominantly vasoconstrictor role. Similarly, while some studies have shown a vasodilator activity of endothelial cyclooxygenase products when measuring skin blood flow in healthy human volunteers (*26*, *27*), others have suggested that the dominant effect is to contribute to vasoconstriction (*28*–*31*).

Where studied, dilator actions of the endothelial cyclooxygenase/prostacyclin pathway have been linked to the IP receptor. While the effects of IP deficiency on blood pressure are inconsistent (*32*, *33*), this pathway has a clear role in protecting against development of vascular disease, including atherosclerosis (*34*). In contrast, the paradoxical vasoconstrictor effects of vascular cyclooxygenase products are mediated by activation of TP (*35*, *36*) or E-prostanoid-3 (EP3) receptors (*10*). These constrictor receptors are commonly expressed in abundance in vascular smooth muscle, whereas dilator IP receptors can be rare/absent (*4*, *37*, *38*). Moreover, TP and EP3 receptors not only can mediate signals to their cognate prostanoid ligands, thromboxane, and prostaglandin E_2_ (PGE_2_) but also can be activated promiscuously by other prostanoids such as PGH_2_, PGF_2α_, and even prostacyclin (*36*, *39*, *40*). When this balance favors activation of constrictor receptors, the result may be not only excessive vascular tone but also development of atherothrombotic disease (*34*, *41*, *42*). We must therefore consider that there may be circumstances where the endothelial cyclooxygenase-1 pathway may be a pathological pathway that should be blocked rather than a protective system that should be preserved.

What remains unclear is whether or not the phenomenon of endothelial cyclooxygenase-1 activity driving vasoconstriction and other pathological responses is meaningful in vivo and contributes to physiological regulation of hemodynamics and/or development of cardiovascular disease. Until recently, there have been no means to answer this because (i) deletion/inhibition of individual prostanoid receptors does not account for the range of prostanoids generated by vascular cyclooxygenase-1 and (ii) global loss of cyclooxygenase-1 does not distinguish between cyclooxygenase-1 products from endothelial cells versus those produced by platelets and other cells. We recently reported results from selective endothelial cyclooxygenase-1 knockout mouse models where the full array of vascular prostanoids is lost, while prostanoid production by platelets is retained and demonstrated that endothelial cycloxygenase-1 protects against thrombosis (*5*). In the current study, we have used these animals to assess the contribution of endothelial prostanoids to vascular function and atherogenesis. In doing so, we provide direct evidence for a pathological role for endothelial cyclooxygenase-1 in vivo and highlight its potential as an unexpected and novel therapeutic target.

## RESULTS AND DISCUSSION

### Eicosanoid formation by isolated arteries; role of cyclooxygenase-1/2 isoforms

**Fig. 1:**
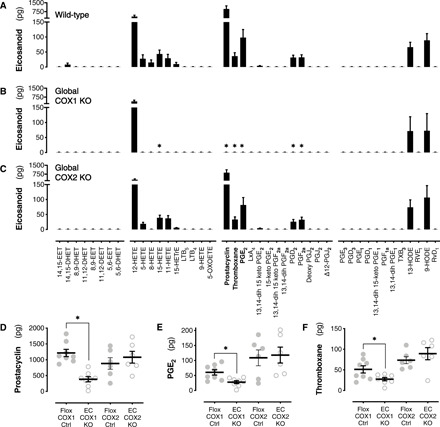
Contribution of endothelial cyclooxygenase isoforms to vascular eicosanoid production. Eicosanoid and related mediator formation from wild-type (**A**), global cyclooxygenase-1 knockout (**B**; COX1 KO), and global cyclooxygenase-2 knockout (**C**; COX2 KO) mouse aorta stimulated with Ca^2+^ ionophore (*n* = 4). Production of prostacyclin (**D**; *n* = 6 to 8), PGE_2_ (**E**; *n* = 6 to 8), and thromboxane (**F**; *n* = 6 to 8) from aorta of endothelial cyclooxygenase-1 or cyclooxygenase-2 knockout mice (EC COX1/COX2 KO) versus respective floxed, Cre-negative littermate controls (Flox COX1/COX2 Ctrl). Prostacyclin and thromboxane were measured as their stable breakdown products, 6-keto-PGF_1α_ and thromboxane B_2_, respectively. Data are means ± SEM. *P < 0.05 versus respective control strain by two-way analysis of variance (ANOVA) with Dunnett’s post-test (A to C) or unpaired *t* test (D to F).

Prostacyclin is generally recognized as the principal cyclooxygenase end-product produced by blood vessels but is generated within a larger family of arachidonic acid metabolites (“eicosanoids”). To understand the breadth of vascular cyclooxygenase-1 metabolites, we first established the full range of eicosanoids produced by isolated vessels from wild-type and cyclooxygenase knockout mice. Wild-type mouse aortae stimulated with calcium ionophore to maximally activate endogenous synthetic pathways released 15 detectable eicosanoid products (Fig. 1A). Within the prostanoid family (eicosanoids generated through cyclooxygenase), prostacyclin was the most abundant, as expected. Vasoconstrictor prostanoids (PGE_2_, PGF_2α_, and thromboxane) were also released, and while this was at a lower level than prostacyclin (5 to 10%), it should be remembered that PGE_2_ and PGF_2α_ have greater stability than prostacyclin and, therefore, an extended duration of action (*38*). 12-hydroxyeicosatetraenoic acid (HETE) was the most abundant nonprostanoid mediator, with lower levels of other HETEs and hydroxyoctadecadienoic acids (HODEs).

Global deletion of cyclooxygenase-1 (from all cell types; *Ptgs1*^−/−^) abolished production of all prostanoid mediators (Fig. 1B), while global deletion of cyclooxygenase-2 (*Ptgs2*^−/−^) had little effect (Fig, 1C). This is consistent with the relative expression of these isoforms in large arteries (*1*) and previous observations that cyclooxygenase-1 is the dominant pathway for prostanoid formation in large mouse arteries regardless of the stimuli (*2*, *3*, *5*). Global cyclooxygenase-1 deletion also prevented release of 15-HETE (with a similar trend apparent for 5-, 8-, and 11-HETEs), in agreement with our previous work in platelets identifying these as cyclooxygenase metabolites (*43*). Little is known about the vascular effects of these mediators; however, in some studies, 5- and 15-HETEs have been identified as vasoconstrictors (*44*), suggesting that they could also contribute to cyclooxygenase-1–driven constrictor responses. Levels of 12-HETE, a product of lipoxygenase activity and the linoleic acid metabolites 9- and 13-HODEs, were unaffected by cyclooxygenase deletion. These data were validated using a pharmacological approach; we found that production of prostacyclin and the vasoconstrictor prostanoid PGE_2_ was reduced from A23187-stimulated aortic rings treated with the selective cyclooxygenase-1 inhibitor SC-560, but not the selective cyclooxygenase-2 inhibitor celecoxib (Table 1). To explore the role of cyclooxygenase-1 in prostanoid generation in microvasculature endothelial cells, these were freshly isolated from wild-type heart and lung using flow cytometric cell sorting to avoid any artifactual changes in cyclooxygenase and synthase expression. Similar to results from aortic endothelial cells, cyclooxygenase-1 was the most abundant isoform at the mRNA level (Table 1A) and both prostacyclin (Table 1B) and PGE_2_ release (Table 1C) by microvascular endothelial cells was sensitive to cyclooxygenase-1 but not to cyclooxygenase-2 inhibition (Table 1). In microvascular endothelial cells from the heart and lung, the balance between prostacyclin and PGE_2_ was shifted (Table 1), with the prostacyclin:PGE_2_ ratio being approximately 2:1 as compared to 15:1 in aortic rings, suggesting that in the microvasculature, the endothelial cyclooxygenase-1 pathway may make an even larger contribution to vasoconstrictor and other pathological responses than in large arteries.

**Table 1:** Effect of cyclooxygenase-1– and cyclooxygenase-2–derived prostaglandin production and expression in aortic rings and microvascular endothelial cells isolated from lung and heart. mRNA expression for cyclooxygenase-1 (Ptgs1) and cyclooxygenase-2 (Ptgs2) in wild-type aortic rings and in microvascular endothelial cells isolated from lung and heart (A), normalized to 18*S* and GAPDH (glyceraldehyde-3-phosphate dehydrogenase) expression for the same cell/tissue sample. Prostacyclin (B; as 6-keto-PGF_1α_) and PGE_2_ formation (C) from the same preparations stimulated with A23187 in the presence of the selective cyclooxygenase-1 inhibitor SC-560 (1 μM), the selective cyclooxygenase-2 inhibitor celecoxib (100 nM), or vehicle (0.1% DMSO). *n* = 3 to 4.

	**Aortic rings**	**Lung EC**	**Heart EC**
A. Gene expression (arbitrary units; relative to 18*S* and GAPDH expression)			
Cyclooxygenase-1	7.9 ± 3.5	128.0 ± 32.6	135.5 ± 32.4
Cyclooxygenase-2	0.6 ± 0.3	6.9 ± 3.1	1.3 ± 0.6
B. Prostacyclin			
Vehicle	50.1 ± 3.8 pg	0.45 ± 0.07 fg/cell	0.46 ± 0.11 fg/cell
SC-560	3.5 ± 0.8 pg*	0.03 ± 0.01 fg/cell*	0.05 ± 0.01 fg/cell*
Celecoxib	53.3 ± 8.6 pg	0.33 ± 0.03 fg/cell	0.76 ± 0.13 fg/cell
C. PGE_2_			
Vehicle	3.6 ± 0.4 pg	0.19 ± 0.03 fg/cell	0.20 ± 0.04 fg/cell
SC-560	0.1 ± 0.0 pg*	0.07 ± 0.01 fg/cell*	0.10 ± 0.04 fg/cell*
Celecoxib	3.6 ± 0.5 pg	0.24 ± 0.06 fg/cell	0.28 ± 0.10 fg/cell

*P < 0.05 versus vehicle by one-way ANOVA with Dunnett’s post-test.

These findings are in keeping with previous work showing that arteries generate a mix of vasodilator and vasoconstrictor prostanoids, which can be removed by physical endothelial disruption (*9*) or global deletion of cyclooxygenase-1 (*2*). However, while these have been useful approaches to understand vascular prostanoids in simple systems, they do not translate to more complex and/or in vivo models where multiple cell types contribute to prostanoid generation. To address this problem, we have previously generated conditional knockout mice where cyclooxygenase-1 is specifically removed from the endothelium, leaving cyclooxygenase-1 products from platelets and other cells intact. These mice show loss of vascular cyclooxygenase-1 immunoreactivity and activity with no compensatory changes in basal or induced cyclooxygenase-2 levels (*5*). Here, we have repeated and extended our previous observations (*5*) to show that prostacyclin (Fig. 1D) as well as the vasoconstrictor prostanoids PGE_2_ (Fig. 1E) and thromboxane (Fig. 1F) are suppressed in aortas from endothelial cyclooxygenase-1 knockout mice (*Ptgs1*^flox/flox^; VE-cadherin-Cre^ERT2^) compared to littermate controls (*Ptgs1*^flox/flox^). By contrast, aortas from endothelial cyclooxygenase-2 knockout mice (*Ptgs2*^flox/flox^; Tie2-Cre) showed no change in production of prostacyclin, PGE_2_, or thromboxane when compared to their own respective littermate controls (*Ptgs2*^flox/flox^). These data not only further confirm endothelial cyclooxygenase-1 as the source of both prostacyclin and vasoconstrictor prostanoids but also validate this model as an appropriate tool to study the role of endothelial prostanoids in cardiovascular physiology and disease.

### Effect of endothelial cyclooxygenase-1/2 deletion on arterial function ex vivo

**Fig. 2:**
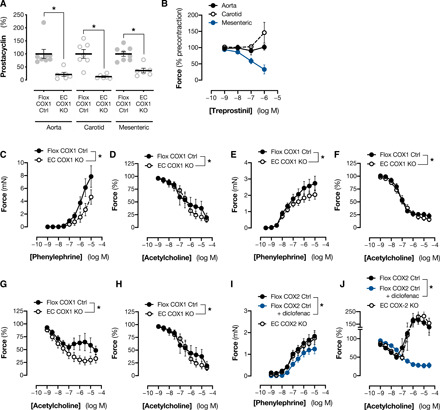
Effect of endothelial cyclooxygenase-1 deletion on contractile and dilator responses in isolated arteries. Prostacyclin (as 6-keto-PGF_1α_) formation from acetylcholine-stimulated arteries (**A**; *n* = 6 to 8) from endothelial cyclooxygenase-1 knockout mice (EC COX1 KO) and floxed littermate controls (Flox COX1 Ctrl). Response to the prostacyclin analog treprostinil in arteries from Flox COX1 Ctrl mice (**B**; *n* = 4 to 5). Vasomotor responses to phenylephrine and acetylcholine in isolated aortae (**C** and **F**; *n* = 6 to 11), mesenteric arteries (**D** and **G**; *n* = 3 to 8), and carotid arteries (**E** and **H**; *n* = 6 to 13) from EC COX1 and respective Flox COX1 Ctrl mice. Responses to phenylephrine (**I**; *n* = 9 to 12) and acetylcholine (**J**; *n* = 10 to 12) in carotid arteries from endothelial cyclooxygenase-2 (EC COX2 KO) mice and respective Flox COX2 Ctrl mice with and without the nonspecific cyclooxygenase-1/2 inhibitor diclofenac (1 μM). Data are means ± SEM. *P < 0.05 by unpaired *t* test (A) or two-way repeated-measures ANOVA with Holm-Sidak post-test (C to J).

We next explored the impact of endothelial cyclooxygenase-1 knockout on the function of isolated vessels, analogous to previous work in the field that highlighted a potential vasoconstrictor role for vascular prostanoids (*4*, *10*, *11*, *18*–*23*). We focused on arteries from three vascular territories—descending aorta, carotid artery, and mesenteric artery—and first studied prostanoid production (measured as prostacyclin). For these experiments, we stimulated vessels with acetylcholine to parallel and interpret protocols including acetylcholine-induced vasodilation reported below. Acetylcholine-induced prostacyclin formation was reduced by >75% by endothelial cyclooxygenase-1 deletion in all vessels (Fig. 2A). However, when exogenous prostacyclin was applied (in the form of its stable analog, treprostinil), only mesenteric arteries showed a dilator response with no response seen in aortae and contraction induced in carotid arteries (Fig. 2B). Therefore, while all three of these vessels have a similar endogenous prostacyclin generation pathway, they capture three distinct phenotypes of prostacyclin responses. To understand the functional role of endogenously generated prostanoids in these vessels, we studied responses to the α_1_-adrenoceptor agonist phenylephrine, which induced concentration-dependent contraction in all three vessels. In each case, this was blunted when cyclooxygenase-1 was selectively deleted from the endothelium (Fig. 2, C to E). A similar effect was produced in aortic rings from wild-type mice treated with the nonselective cyclooxygenase-1/2 inhibitor diclofenac in tissues contracted by phenylephrine 5-hydroxytryptamine (5-HT), PGE_2_, or the thromboxane mimetic U46619 (fig. S1), confirming that the effect is not an artifact of genetic modification or a specific interaction between cyclooxygenase and adrenoceptor signaling. Instead, these findings are consistent with the idea that endogenous prostanoids in each of these vessels contribute to contractile tone and reduce the threshold for vasoconstriction. A constrictor role for endothelial cyclooxygenase-1 was seen even in the mesenteric artery where prostacyclin acts as a dilator in vessels from both floxed control and endothelial cyclooxygenase-1 knockout mice (Fig. 2B and fig. S2), suggesting that it is mediated by other endothelium-derived prostanoids such as PGE_2_. When the endothelium was specifically activated with acetylcholine in precontracted vessels, dilation was evoked in all three arteries (Fig. 2, F to H). In carotid arteries, but not aortae or mesenteric arteries, the response to acetylcholine was biphasic, characterized by dilation at lower concentrations and contraction at higher concentrations (>100 nM). Deletion of cyclooxygenase-1 from the endothelium had no effect on acetylcholine-induced dilation in any vessel (Fig. 2, F to H) but abolished the contractile response to higher concentrations of acetylcholine seen in the carotid artery (Fig. 2H), again in agreement with the idea that overall endothelium-derived prostanoids drive vasoconstrictor responses. To confirm that this role was specifically associated with endothelial cyclooxygenase-1 activity, studies of carotid artery function were repeated in vessels from endothelium-specific cyclooxygenase-2 knockout mice. In agreement with the minimal contribution of cyclooxygenase-2 to vascular prostanoid production, carotid arteries from these mice demonstrated no alteration in the responses to phenylephrine (Fig. 2I) or acetylcholine (Fig. 2J) compared to their respective controls but the inhibitory effect of endothelial cyclooxygenase-1 deficiency could be replicated in vessels from these animals by pharmacological cyclooxygenase inhibition with diclofenac (Fig. 2, I and J).

### Effect of endothelial cyclooxygenase-1/2 deletion on hemodynamic responses in vivo

**Fig. 3:**
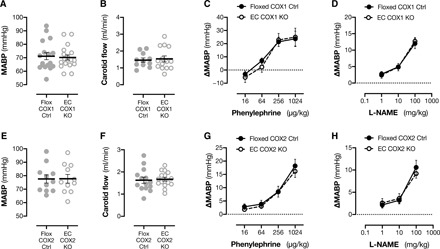
Effect of endothelial cyclooxygenase-1 and cyclooxygenase-2 deletion on hemodynamic responses in vivo. Mean arterial blood pressure (MABP) (**A**; *n* = 17 to 18) and carotid artery blood flow (**B**; *n* = 11 to 14) in anesthetized endothelial cyclooxygenase-1 knockout mice (EC COX1 KO) versus floxed littermate controls (Flox COX1 Ctrl). Cumulative dose-blood pressure response curves to intravenous phenylephrine (**C**; *n* = 4 to 10) and L-NAME (**D**; *n* = 7 to 12) in EC COX1 KO and Flox COX1 Ctrl mice. Mean arterial blood pressure (**E**; *n* = 11) and carotid artery blood flow (**F**; *n* = 14 to 16) in anesthetized endothelial cyclooxygenase-2 knockout mice (EC COX2 KO) and floxed littermate controls (Flox COX2 Ctrl). Cumulative dose-blood pressure response curves to intravenous phenylephrine (**G**; *n* = 3) and L-NAME (**H**; *n* = 5) in EC COX2 KO and Flox COX2 Ctrl mice. Data are means ± SEM. All *P* > 0.05 by unpaired *t* test (A, B, E, and F) or two-way repeated-measures ANOVA (C, D, G, and H).

Our results from isolated vessels fully support the concept that the net action of endothelial cyclooxygenase-1–derived prostanoids on arterial function is to drive vasoconstriction. The mechanisms for this have been explored extensively elsewhere and likely result from activation of vasoconstrictor TP and EP3 receptors (*4*, *10*, *35*, *36*, *38*, *45*), but the consequence of this phenomenon to cardiovascular function in vivo has not been addressed. Certainly, any effects of global deletion of cyclooxygenase-1 (*19*, *46*) or the individual constrictor/dilator prostanoid receptor IP (*33*), EP3 (*47*), or TP (*48*) on basal hemodynamics are minimal. However, given the complex multireceptor pharmacology of endothelium-derived prostanoids, and the production of prostanoids with competing biological activity by cyclooxygenase-1 in different cell types, it is not possible to infer the overall contribution of endothelium-derived prostanoids by these approaches. Consequently, we studied hemodynamic responses in vivo in endothelial cyclooxygenase-1 knockout mice, where the full repertoire of endothelial prostanoids is removed, while those produced by other cells such as platelets and the kidney remain intact. In these animals, under anesthesia, mean arterial blood pressure (Fig. 3A), heart rate [Flox COX1 Ctrl, 483 ± 12 beats per minute (BPM); EC COX1 KO, 478 ± 14 BPM; *n* = 11 to 13, *P* = 0.79 by unpaired *t* test], and carotid artery blood flow (Fig. 3B) were not different from those in floxed controls. To model the effects observed on phenylephrine-induced contraction of isolated vessels in vitro, we examined the pressor response to phenylephrine in vivo and found that this response was also unaltered by endothelial cyclooxygenase-1 deletion (Fig. 3C). We next determined whether an effect of endothelial prostanoid generation on blood pressure regulation was masked by endothelial nitric oxide production; we measured mean arterial pressure in the presence of increasing concentrations of the nitric oxide synthase inhibitor L-NG-Nitro arginine methyl ester (L-NAME). However, while nitric oxide synthase inhibition increased blood pressure as expected, this response was not different when endothelial cyclooxygenase-1 was deleted (Fig. 3D). Last, because endothelial cyclooxygenase-2 limits thrombosis (*5*, *6*), we considered its contribution to vascular responses in vivo. In agreement with previous reports (*6*), endothelial cyclooxygenase-2 deletion had no effect on resting blood pressure (Fig. 3E). Endothelial cyclooxygenase-2 knockout mice also displayed normal heart rate (Flox COX2 Ctrl, 517 ± 12 BPM; EC COX2 KO, 526 ± 8 BPM; *n* = 14 to 16, *P* = 0.52 by unpaired *t* test), carotid flow (Fig. 3F), and pressor responses to phenylephrine (Fig. 3G) and L-NAME (Fig. 3H), excluding a role of endothelial cyclooxygenase-2 in baseline hemodynamic control. Overall, therefore, these data demonstrate that despite the vasoconstrictor role of endothelial prostanoids observed in isolated vessel bioassays, local generation of prostanoids within the endothelium does not meaningfully contribute to systemic basal vascular tone or hemodynamic control in vivo. It cannot be excluded, however, that there may be a role for vascular prostanoids in local organ blood flow that is not represented in systemic hemodynamic parameters and/or that a role may emerge in situations of cardiovascular disease such as hypertension where vessel contractility and endothelial constrictor prostanoids are augmented.

### Effect of endothelial cyclooxygenase-1 deletion in atherosclerosis

With little apparent effect on basal cardiovascular physiology, we next considered whether the activation of “constrictor” prostanoid receptors by endothelial cyclooxygenase-1 products might contribute to the development of more complex cardiovascular pathologies. We chose to study the development of atherosclerosis using a standard high-fat/cholesterol-fed ApoE^−/−^ model because these mice are sensitive both to the protective effects of prostacyclin (*34*) and to the detrimental effects of constrictor prostanoid receptor activation (*34*, *41*, *42*). A negative role for cyclooxygenase-1 in this model has been previously inferred from studies with global cyclooxygenase-1 deletion or inhibition (*49*, *50*) and has been typically attributed to the loss of the platelet cyclooxygenase-1–derived thromboxane. Intriguingly, however, Babaev *et al.* (*51*) showed that deletion of cyclooxygenase-1 from bone marrow, which resulted in complete loss of platelet cyclooxygenase-1 activity, failed to alter atherosclerotic disease burden, indicating that the pro-atherogenic role of cyclooxygenase-1 may also be associated with expression in stromal cells. To study the role of endothelial prostanoids in this process, we crossed our endothelial cyclooxygenase-1 knockout mice onto an ApoE-deficient background and induced atherosclerotic plaque formation with high-fat/cholesterol feeding.

**Fig. 4:**
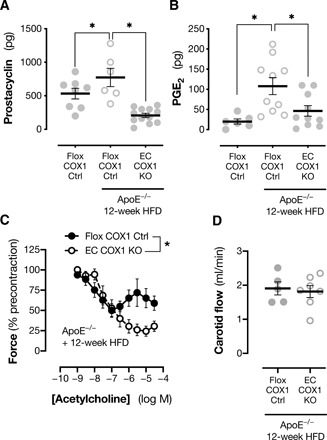
Effect of endothelial cyclooxygenase-1 deletion on prostanoid formation and vascular function in atherosclerotic animals. Prostacyclin (**A**; *n* = 6 to 12; as 6-keto-PGF_1α_) and PGE_2_ formation (**B**; *n* = 6 to 10) from A23187-stimulated carotid arteries from healthy and atherosclerotic [ApoE^−/−^; 12-week high-fat diet (HFD)/high-cholesterol diet] endothelial cyclooxygenase-1 knockout mice (EC COX1 KO) and floxed littermate controls (Flox COX1 Ctrl). Vasomotor responses to acetylcholine in isolated carotid arteries ex vivo (**C**; *n* = 6 to 8) and carotid artery blood flow under anesthesia in vivo (**D**; *n* = 5 to 7) in atherosclerotic EC COX1 KO and Flox COX1 Ctrl mice. Data are means ± SEM. *P < 0.05 by (A and B) one-way ANOVA with Holm-Sidak post-test, two-way repeated-measures ANOVA (C) or unpaired *t* test (D).

We first examined prostanoid formation, stimulated with calcium ionophore (as in Fig. 1), to maximally activate endogenous biosynthetic enzyme pathways in carotid arteries from atherosclerotic animals (*ApoE*^−/−^; *Ptgs1*^flox/flox^) and healthy animals (*Ptgs1*^flox/flox^). We observed a modest (~1.5-fold) increase in prostacyclin production (Fig. 4A) and a larger (~4-fold) increase in PGE_2_ production (Fig. 4B) in diseased vessels. This was not associated with significant changes in mRNA for the inflammatory PGE_2_ synthase mPGES-1 (table S1), suggesting that the shift toward PGE_2_ production was a result of changes in other PGE_2_ synthetic pathways and/or altered cellular composition. Cyclooxygenase-2 expression was significantly increased in atherosclerotic vessels (table S1), but cyclooxygenase-1 remained the dominant isoform at the mRNA level, consistent with our previous observations of cyclooxygenase immunoreactivity in ApoE-deficient mice (*52*). Accordingly, release of both prostacyclin (Fig. 4A) and PGE_2_ (Fig. 4B) was strongly attenuated by endothelial cyclooxygenase-1 deletion (*ApoE*^−/−^; *Ptgs1*^flox/flox^; VE-cadherin-Cre^ERT2^).

**Fig. 5:**
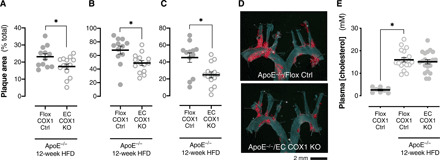
Effect of endothelial cyclooxygenase-1 deletion on aortic and circulating lipid accumulation in atherosclerotic animals. Aortic lipid accumulation in the aortic arch (**A**; *n* = 12 to 14), the brachiocephalic artery (**B**; *n* = 12 to 14), and carotid artery (**C**; *n* = 12 to 14) from atherosclerotic (ApoE^−/−^; 12-week high-fat/cholesterol diet) endothelial cyclooxygenase-1 knockout mice (EC COX1 KO) and floxed littermate control mice (Flox COX1 Ctrl) and representative photographs of sudan IV–stained aortic arches from each strain (**D**). Plasma total cholesterol levels from healthy and atherosclerotic EC COX1 KO and Flox COX1 Ctrl mice (**E**; *n* = 8 to 21). Data are means ± SEM. *P < 0.05 by unpaired *t* test (A to C) or one-way ANOVA with Holm-Sidak post-test (E).

When we analyzed the function of atherosclerotic carotid arteries, the impact of endothelial cyclooxygenase-1 deletion was similar to that seen in healthy vessels: Vasoconstrictor responses to phenylephrine were blunted (*E*_max_; Flox COX1 Ctrl, 2.46 ± 0.27 mN; EC COX1 KO, 1.87 ± 0.27 mN; *P* < 0.01 by *F* test to compare curve fit; *n* = 6 to 7), and the constrictor responses to high concentrations of acetylcholine were prevented (Fig. 4C). These data suggest that in atherosclerotic animals just as in healthy animals, the dominant activity of vascular prostanoids is to act on constrictor receptors such as TP and EP3. To understand the impact of this in vivo, we again studied hemodynamic responses and found no effect of endothelial cyclooxygenase-1 deletion on carotid artery blood flow (Fig. 4D), blood pressure (Flox COX1 Ctrl, 71.8 ± 3.0 mmHg; EC COX1 KO, 71.2 ± 2.8 mmHg; *P* = 0.89 by unpaired *t* test), or heart rate (Flox COX1 Ctrl, 508 ± 35 BPM; EC COX1 KO, 507 ± 17 BPM; *P* = 0.99 by unpaired *t* test). However, when we examined the atherosclerotic disease severity in these animals, we found that endothelial cyclooxygenase-1 deletion limited plaque formation in the aortic arch (Fig. 5A) and its major branches (Fig. 5, B to D) without altering circulating levels of cholesterol (Fig. 5E). Therefore, in this model, despite prostacyclin being the most abundant prostanoid generated by endothelial cyclooxygenase-1, the net effect of the full range of endothelial cyclooxygenase-1–derived prostanoids is to augment the development of atherosclerotic disease. This result is consistent with the idea that, although IP receptor activation can be protective in this model (*34*), IP receptor–mediated protection is overshadowed by activation of pathogenic, constrictor prostanoid receptors such as TP and EP3, by prostacyclin itself, or by other endothelium-derived prostanoids such as PGE_2_ and thromboxane.

**Fig. 6:**
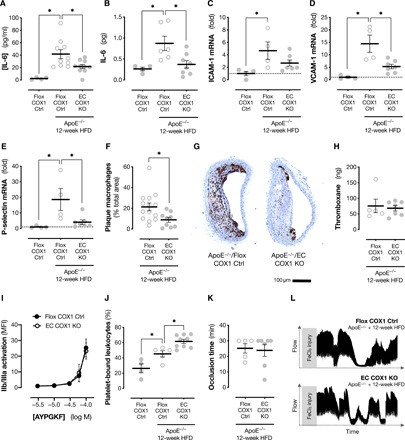
Effect of endothelial cyclooxygenase-1 deletion on inflammatory and platelet function in atherosclerotic animals. Levels of IL-6 in plasma (**A**; *n* = 4 to 10) and released from isolated carotid artery (**B**; *n* = 4 to 8) from healthy and atherosclerotic (ApoE^−/−^; 12-week high-fat/cholesterol diet) endothelial cyclooxygenase-1 knockout mice (EC COX1 KO) and floxed littermate control mice (Flox COX1 Ctrl). Expression of mRNA for ICAM-1 (**C**), VCAM-1 (**D**), and P-selectin (**E**) in aortic arches from healthy and atherosclerotic EC COX1 KO and Flox COX1 Ctrl mice. Macrophage (Mac2^+^ cell) immunoreactivity in brachiocephalic artery sections from atherosclerotic EC COX1 KO and Flox COX1 Ctrl mice (**F**) and representative micrographs of staining in each genotype (**G**). Thromboxane generation from A23187-stimulated whole blood from atherosclerotic EC COX1 KO and Flox COX1 Ctrl mice (**H**). AYGKPF (a thrombin mimetic peptide)–induced platelet GPIIb/IIIa activation (**I**; *n* = 5 to 10) and basal circulating platelet-leukocyte binding (**J**; *n* = 4 to 9) in whole blood from healthy and atherosclerotic EC COX1 KO and Flox COX1 Ctrl animals. Thrombosis in vivo measured as time to occlusion after FeCl_3_-induced injury to the carotid artery in atherosclerotic EC COX1 KO and Flox COX1 Ctrl mice (**K**; *n* = 5 to 7) and representative flow traces from each genotype (**L**). Data are means ± SEM. *P < 0.05 by one-way ANOVA with Holm-Sidak post-test (A to E and J), unpaired *t* test (F, H, and K), or two-way repeated-measures ANOVA (I).

Prostanoids have a complex biology, positively and negatively modulating many processes that underlie atherosclerotic disease development. Among these, reductions in vascular inflammation and platelet activation have previously been implicated in the reduced atherothrombosis observed in mouse models of global cyclooxygenase-1, TP, and EP3 receptor deletion/inhibition. To understand mechanistically the reduction in atherogenesis observed in endothelial cyclooxygenase-1–deficient mice, we focused on these processes. Atherosclerotic animals showed evidence of systemic and local vascular inflammation with elevated plasma levels of interleukin-6 (IL-6; Fig. 6A), increased IL-6 release from isolated carotid arteries (Fig. 6B), and up-regulation of the adhesion molecules intercellular adhesion molecule–1 (ICAM-1; Fig. 6C), vascular cell adhesion molecule–1 (VCAM-1; Fig. 6D), and P-selectin in the aortic arch (Fig. 6E). Plasma and carotid IL-6 levels and aortic arch expression of VCAM-1 and P-selectin were strongly reduced by endothelial cyclooxygenase-1 deletion, with a similar nonsignificant trend noted for ICAM-1 expression (Fig. 6, A to F). In agreement, when we examined the brachiocephalic artery histologically, we observed reduced infiltration of macrophages (Mac2 immunoreactive cells) in plaques from endothelial cyclooxygenase-1–deficient mice (Fig. 6, F and G), consistent with an anti-inflammatory, stabilized plaque phenotype. These results are consistent with the idea that endothelial prostanoids may drive atherogenesis by activating TP/EP receptors to up-regulate vascular cytokines/adhesion molecules, resulting in increased inflammatory cell infiltration and plaque growth. These results mirror responses seen in ApoE^−/−^ mice where TP is deleted/inhibited in which reduced lesion formation is accompanied by reductions in circulating IL-6 (*53*), tumor necrosis factor–α (TNFα) (*53*), sICAM-1 (*54*), and endothelial ICAM-1 immunoreactivity (*34*).

In parallel, we assessed the impact of endothelial cyclooxygenase-1 deletion on platelet responses in our atherosclerotic mice. As endothelial cyclooxygenase-1 knockout mice were generated using a VE-cadherin Cre^ERT2^ driver, which produces a highly selective vascular deletion, platelet cyclooxygenase-1 activity assessed by thromboxane release was unaffected as expected (Fig. 6H). Similarly, ex vivo platelet activation to the thrombin-mimetic peptide AYPGKF measured as glycoprotein (GP) IIb/IIIa activation (Fig. 6I) and P-selectin expression [half-maximal effective concentration (EC_50_); Flox COX1 Ctrl, −4.12 ± 0.08; EC COX1 KO, −4.10 ± 0.06; *P* = 0.80 by *F* test to compare curve fits] was unaltered in these animals. While these results above show that the anti-atherogenic effect of endothelial cyclooxygenase-1 deletion is not due to inadvertent loss of platelet reactivity or cyclooxygenase-1 activity, as ex vivo assays, they do not consider how the platelet acts in vivo where it is exposed to short-lived endothelium-derived prostanoids. To address this question, we measured levels of platelet adhesion to leukocytes as a marker of platelet reactivity in the body. Circulating levels of platelet-bound leukocytes were increased in atherosclerotic compared to healthy animals, reflecting higher basal level of platelet activation in the diseased state (Fig. 6J). This was significantly augmented in endothelial cyclooxygenase-1 knockout mice, suggesting that loss of endothelial prostanoids increases circulating platelet reactivity. While increased platelet reactivity in vivo is unlikely to explain the observed reduction in atherosclerosis in these animals, it reflects prostacyclin’s anti-thrombotic importance and dominance in the regulation of platelet reactivity by endothelial prostanoids. To explore this question further, we used an in vivo ferric chloride thrombosis model to study the impact of endothelial cyclooxygenase-1 on thrombosis in atherosclerotic mice. Unexpectedly, and in contrast to what we have previously seen in healthy animals (*5*), in atherosclerotic carotid arteries, endothelial cyclooxygenase-1 deletion had no effect on thrombotic occlusion time (Fig. 6, K and L). Although we cannot exclude that this negative result reflects the difficulty in modeling of atherothrombosis in mice, it may suggest that the normal anti-thrombotic activity of the endothelial prostacyclin pathway (*5*) is lost in this setting, either because of the increased thrombogenicity of the diseased vessel or because accumulation of stable pro-thrombotic prostanoids such as PGE_2_ within plaques overrides any anti-thrombotic effect of short-lived prostacyclin (*41*).

### Potential of vascular cyclooxygenase-1 as a cardiovascular therapeutic target

These results demonstrate that endothelial prostanoids provide meaningful activation of pathological signaling pathways in vivo and cyclooxygenase-1 products from endothelial cells, along with those released from platelets, have an active role in the development of cardiovascular disease. Inhibition of platelet cyclooxygenase-1 by low-dose aspirin is an important therapeutic strategy to reduce cardiovascular risk, but in light of these results, we must now consider whether simultaneous inhibition of endothelial cyclooxygenase-1 might improve rather than diminish the cardiovascular protection provided by aspirin-like therapies. While we must, of course, be cautious of extrapolating data from mouse models in humans, the findings presented here suggest that adding blockade of endothelial cyclooxygenase-1 would further protect against atherogenesis, vascular inflammation, and perhaps, in some cases, excessive vasoconstriction.

The question remains whether this would be outweighed by the loss of anti-thrombotic activity of cyclooxygenase-1–derived prostacyclin. In this light, our observations that endothelial cyclooxygenase-1 deletion has no effect on thrombosis at sites of atherosclerotic plaques may minimize such concerns. In addition, even in healthy animals, we previously found that while endothelial cyclooxygenase-1 deletion increases thrombosis, when cyclooxygenase-1 is deleted from both platelets and endothelial cells, the endothelial phenotype is overridden and the net effect is firmly anti-thrombotic (*5*). Further, in some animal models, global cyclooxygenase-1 deletion/inhibition, which removes both platelet and endothelial prostanoids, still reduces thrombosis (*55*, *56*) while also protecting against atherosclerosis (*49*, *50*) and hypertension (*57*) and is consistent with an overall “bleeding” phenotype in patients with putative cyclooxygenase-1 deficiency (*58*, *59*). Perhaps due to previous dogma concerning the roles of cyclooxygenase-1 and cyclooxygenase-2 in the cardiovascular system, the potential cardioprotective effects of global cyclooxygenase-1 inhibition have not been evaluated clinically. Candidate drugs such as ketorolac, which is >100-fold cyclooxygenase-1 selective (*60*), exist but are not currently used at cyclooxygenase-1–selective doses. Any cardiovascular protective therapy based on cyclooxygenase-1 inhibition would need to be carefully designed to avoid the cardiovascular damage associated with unintentional cyclooxygenase-2 inhibition (*61*) and the gastrointestinal damage that arises when both cyclooxygenase-1 and cyclooxygenase-2 are inhibited concurrently (*62*). However, if applied correctly, pan–cyclooxygenase-1 inhibitors or drugs targeting specific pathological prostanoid receptors might have potential not only in the management of atherothrombotic disease but also in other conditions, including acute pain and some cancers (*63*), where cyclooxygenase-1–derived prostanoids play a pathological role.

### Conclusions

Overall, these studies show that while endothelial cyclooxygenase-1 is the dominant driver of local vascular prostacyclin, with all implications of cardioprotection that that observation brings, cyclooxygenase-1–driven products can also drive vasoconstriction in vitro and atherogenesis in vivo. This does not mean that endothelial cyclooxygenase-1 activity is detrimental in all circumstances; endothelial cyclooxygenase-1–derived prostacyclin can be protective in other settings such as thrombosis, just as cyclooxygenase-2 can exert both protective (*5*, *6*, *52*, *61*) and harmful (*64*, *65*) effects on cardiovascular health depending on the context. Instead, this dichotomy emphasizes that to understand the roles of cyclooxygenase enzymes and their products in cardiovascular health, we need to consider the full breadth of prostanoids generated, the abundance of their receptors on target tissues, and the specific pathological context. With this in mind, the findings of the current study support the case that, in the setting of atherogenesis, endothelial cyclooxygenase-1 should be considered a therapeutic target. This must now be fully explored in animal models and in humans to evaluate the potential of “super-aspirin” drugs that inhibit cyclooxygenase-1 globally (including both platelets and endothelial cells) to provide additional cardioprotective benefit.

## MATERIALS AND METHODS

### Animals

All procedures were carried out in accordance with Animals (Scientific Procedures) Act (1986) Amendment Regulations (2012) after review by the Imperial College Ethical Review Board and the authority of UK Home Office Project licenses 70/8422 and PP1576048. Animals were housed in individually ventilated cages with free access to food and water and a 12-hour day/night cycle. Unless otherwise indicated, experiments were performed on male and female 8- to 12-week-old mice. Animals were randomized through allocation of sequential number before genotyping, and experiments were performed in this order. All data analysis was conducted by an operator blinded to the genotype.

Five sets of animals were used in these studies. First were global cyclooxygenase-1 knockout mice, which were generated as previously described (*66*), that carry complete loss of cyclooxygenase-1 activity across all cells in the body (Fig. 1). Second were global cyclooxygenase-2 mice, also as previously described (*67*), that carry complete loss of cyclooxygenase-2 activity across all cells in the body (Fig. 2). Both these strains were maintained on a pure C57Bl/6 background and compared to wild-type C57Bl/6 age- and sex-matched nonlittermate controls. Third were endothelial cyclooxygenase-1 knockout mice (Ptgs1^flox/flox^ VE-cadherin-Cre^ERT2^) that were generated by crossing floxed *Ptgs1* mice with VE-cadherin Cre^ERT2^ mice as we have recently described (*5*) (Figs. 1 to 3). These mice carry selective loss of cyclooxygenase-1 only from vascular endothelial cells and were maintained on a C57Bl/6 background. They were compared to littermate controls (“Flox COX1 Ctrl”) that carry a floxed *Ptgs1* allele but without a Cre transgene and therefore retain full cyclooxygenase activity. The Cre^ERT2^ construct used in these animals is silent until activated by tamoxifen, and therefore, these animals were treated with tamoxifen at age 5 to 6 weeks for 5 days (50 mg/kg, intraperitoneally, once daily; Sigma-Aldrich, UK) to induce endothelial cyclooxygenase-1 deletion and then allowed to recover for at least 2 weeks before carrying out experiments. Fourth were endothelial cyclooxygenase-1 knockout mice (as above) crossed onto a homozygous ApoE-deficient background (ApoE^−/−^ Ptgs1^flox/flox^ VE-cadherin-Cre^ERT2^) to increase susceptibility to atherosclerosis (Figs. 4 to 6). These animals were compared to homozygous ApoE-deficient littermates carrying the floxed *Ptgs1* allele but without a Cre transgene and therefore have the same potential for atherosclerosis but retain full cyclooxygenase activity. Fifth were endothelial cyclooxygenase-2 knockout mice (Ptgs2^flox/flox^ Tie2-Cre) generated by crossing floxed *Ptgs2* mice with Tie2-Cre^ERT2^ mice as we have recently described (*5*) (Figs. 1 to 3). These mice carry selective loss of cyclooxygenase-2 only from vascular endothelial cells and were maintained on a mixed C57Bl/6 × 129S4/SvJae background. They were compared to littermate controls (“Flox COX2 Ctrl”) that carry a floxed *Ptgs2* allele but without a Cre transgene and therefore retain full cyclooxygenase activity.

### Vascular eicosanoid and cytokine release

Aortae, carotid, and mesenteric arteries were isolated from mice euthanized by CO_2_ narcosis or isoflurane overdose and cleaned of peri-adventitial material and divided into 2-mm rings. Arterial rings were allowed to equilibrate in phosphate-buffered saline (PBS) at room temperature for 30 min, then transferred to microtiter plate wells containing Dulbecco’s modified Eagle’s medium (Sigma-Aldrich, UK) and acetylcholine (10 μM; Sigma-Aldrich, UK) or A23187 (30 μM; Sigma-Aldrich, UK), and incubated for 30 min at 37°C with gentle shaking (BioShake IQ, Q Instruments, Germany). Where indicated, the selective cyclooxygenase-1 inhibitor SC-560 (1 μM; Abcam, UK), the selective cyclooxygenase-2 inhibitor celecoxib (100 nM; Sigma-Aldrich, UK), or vehicle [0.1% dimethyl sulfoxide (DMSO); Sigma-Aldrich, UK] was added to both the preincubation and stimulation media. Supernatants were collected for measurement by commercial immunoassay kits for prostacyclin (as its stable breakdown product, 6-keto-PGF_1α_; Cayman Chemical, USA), thromboxane (as its stable breakdown product, TXB_2_; Cayman Chemical, USA), PGE_2_ (Cisbio, France), and IL-6 (BioLegend, Germany). In some cases, a full eicosanoid array was measured using liquid chromatography–tandem mass spectrometry as we have described (*5*, *68*).

### Microvascular endothelial cell isolation and prostanoid release

Heart and lung from wild-type mice were finely minced and then digested using collagenase I (5 mg/ml; Sigma-Aldrich, UK), elastase (100 μg/ml; Sigma-Aldrich, UK), and deoxyribonuclease I (125 U/ml; Sigma-Aldrich, UK) at 37°C for 45 min with regular mixing. The resulting cell suspension was strained, and red cells were lysed using Ammonium-Chloride-Potassium buffer (Gibco, UK). Cells were incubated with rat anti-mouse CD31–peridinin chlorophyll protein/cyanine 5.5 (PerCP/Cy5.5; 2.5 μg/ml; clone MEC13.3; BioLegend, UK) and rat anti-mouse CD45–phycoerythrin/cyanine 7 (PE/Cy7; 2.5 μg/ml; clone 30-F11; BioLegend, UK) for 10 min in the presence of TruStain FcX CD16/32 receptor block (10 μg/ml; clone 93; BioLegend, UK) and then resuspended in 4′,6-diamidino-2-phenylindole (DAPI; 5 μg/ml; Life Technologies, UK). Stained cell suspensions were sorted using a FACSAria III instrument (BD Biosciences, Germany). Subcellular debris, doublets, and dead cells were excluded on the basis of forward and side scatter, and DAPI parameters, and then CD31^+^ and CD45^−^ events were sorted using a 100-μm nozzle in purity sort mode. Sorted cells were incubated with vehicle (0.1% DMSO), SC-560 (1 μM), or celecoxib (100 nM) for 30 min at 37°C before addition of A23187 (30 μM) for a further 30 min. Cell supernatants were collected for measurement of prostacyclin and PGE_2_ by immunoassay as above, and cell pellets were retained for RNA extraction. At all stages, buffers contained the protein synthesis inhibitor cycloheximidie (3 μM; Sigma-Aldrich, UK).

### In vitro vascular function

Vascular responses in isolated arteries prepared as above were studied by wire myography. Artery rings were mounted in organ baths connected to isometric force transducers in a Danish Myo Technology (Denmark) 610M myography in Krebs buffer (composition: 120 mM NaCl, 4.7 mM KCl, 1.2 mM MgSO_4_, 1.2 mM KH_2_PO_4_, 25 mM NaHCO_3_, 30 μM EDTA, and 5.5 mM d-glucose; all from Sigma-Aldrich, UK). A basal tension of 8 mN was applied to all vessels, and then their viability was assessed by measuring contraction to KCl (125 mM; Sigma-Aldrich, UK). Cumulative response curves to the contractile agent phenylephrine (Sigma-Aldrich, UK), 5-HT (Sigma-Aldrich, UK), U46619 (Cayman Chemical, USA), PGE_2_, or 5-HT (Sigma-Aldrich, UK) were recorded. After washing, vessels were precontracted with an EC_50_ of phenylephrine and then cumulative response curves to acetylcholine (Sigma-Aldrich, UK) or treprostinil (R&D Systems, UK) were constructed. Where indicated, vessels were treated with diclofenac (1 μM; Sigma-Aldrich, UK) for 10 min before measuring the response under study. Data were expressed as absolute force developed (contractile responses) or as a percentage of the precontraction (dilator responses). If vessels failed to give adequate relaxation to acetylcholine (aortae/mesenteric arteries, >50%; carotid arteries, >25%), data from those vessels were excluded on the basis of endothelial damage during isolation.

### In vivo hemodynamics

Under isoflurane anesthesia, the left carotid artery was cannulated with PE10 tubing connected to a physiological pressure transducer (AD Instruments, UK). Basal arterial pressure was recorded before measuring a cumulative dose-response curve to phenylephrine (Sigma-Aldrich, UK) or L-NAME (Sigma-Aldrich, UK), administered via the jugular vein. In separate animals, a Doppler flow probe (Transonic Systems, USA) was placed around the left carotid artery to measure basal carotid blood flow and to derive heart rate.

### Atherosclerosis, lipid, and cytokine measurements

Atherosclerosis was induced by administration of a high-fat (21%)/high-cholesterol (1.25%) diet (D12108C; Research Diets, USA) to mice on a homozygous ApoE-deficient background. After 12 weeks, mice were euthanized by CO_2_ narcosis, blood was collected into heparin (10 U/ml; Leo Laboratories, UK), and the vascular tree was perfused via the heart with PBS and formalin. The descending aorta, aortic arch, and its major branches were carefully dissected, opened for en face lipid staining with sudan IV (Sigma-Aldrich, UK), and imaged using a Stemi 305 stereomicroscope (Carl Zeiss, Germany). The percentage area of each vessel stained with sudan IV was quantified using Photoshop 21.1.2 software (Adobe Systems, USA). Plasma was separated from whole blood by centrifugation, and levels of total cholesterol (Cell Biolabs, USA) and IL-6 (BioLegend, Germany) were measured using commercial assay kits.

### Immunohistochemistry

Brachiocephalic arteries from atherosclerotic mice were fixed in 10% neutral-buffered formalin (Sigma-Aldrich, UK) for 48 hours and then infiltrated/embedded in paraffin. Four-micrometer serial sections at 50-μm intervals were cut through the length of the artery, and regions containing plaques were selected for analysis. Sections were rehydrated, blocked with 3% H_2_O_2_ (Sigma-Aldrich, UK) and then 20% goat serum (Abcam, UK) + 1% bovine serum albumin (BSA) in PBS (Sigma-Aldrich, UK), and incubated in rat anti-mouse Mac2 primary antibody (clone M3/38; 0.2 μg/ml; 18 hours; BioLegend, UK). With extensive washing between, sections were then incubated with biotinylated goat anti-rat immunoglobulin G (IgG) secondary antibody (2.8 μg/ml; 30 min; Life Technologies; UK) and streptavidin-labeled peroxidase (1:200; 30 min; Sigma-Aldrich; UK). Antibody complexes were visualized using 3,3-diaminobenzidine (Vector Labs; UK), and nuclei were counterstained using filtered hematoxylin (Sigma-Aldrich, UK). Stained sections were dehydrated, mounted, and imaged under a 10× objective magnification using an AxioVert.A1 microscope (Carl Zeiss, Germany).

### Quantitative polymerase chain reaction

Cells or aortic arches were isolated as above and then homogenized in RLT buffer (Qiagen, UK) + 1% β-mercaptoethanol (Sigma-Aldrich, UK). RNA was extracted from crude homogenates using RNeasy Mini kits (Qiagen, UK). TaqMan assays were used to measure expression of cyclooxygenase-1 (*Ptgs1*; assay ID: Mm00477214_m1), cyclooxygenase-2 (*Ptgs2*; assay ID: Mm00478374_m1), mPGES-1 (*Ptges*; assay ID: Mm00452105_m1), ICAM-1 (*Icam1*; assay ID: Mm00516023_m1), VCAM-1 (*Vcam1*; assay ID: Mm01320970_m1), or *Selp* (assay ID: Mm00441295_m1) using a 1-step RT-qPCR master mix (Promega, UK) and 7500FAST instrument (Applied Biosystems, USA). Expression was normalized against the housekeeping genes 18S (*18s*; assay ID: Hs99999901_s1) and GAPDH (glyceraldehyde-3-phosphate dehydrogenase) (*Ptges*; assay ID: Mm99999915_g1) using the comparative *C*_t_ method.

### Platelet activation and thrombosis

Platelet thromboxane formation in vitro was stimulated in whole blood stimulated with A23187 (30 μM; Sigma-Aldrich, UK) at 37°C for 30 min and measured with a commercial immunoassay kit (Cayman Chemical, USA). Platelet activation in vitro and in vivo was measured by flow cytometry using an LSR Fortessa instrument (BD Biosciences, USA). For in vitro platelet stimulation, whole blood diluted 1:20 in Tyrodes buffer was stimulated with the thrombin receptor–mimetic AYGKPF (an agonist for the PAR4 receptor) for 10 min at room temperature in the presence of the following antibodies: rat anti-mouse activated GPIIb/IIIa-PE (clone JON/A; 1:10; emfret Analytics, Germany), rat anti-mouse P-selectin–fluorescein isothiocyanate (clone Wug.E9; 1:10; emfret Analytics, Germany), and rat anti-mouse CD41–allophycocyanin (APC clone MWReg30; 5 μg/ml; BioLegend, UK). For in vivo platelet/leukocyte adhesion, undiluted, unstimulated whole blood was labeled with rat anti-mouse CD41-allophycocyanin for 10 min. In each case, antibodies were incubated for 10 min at room temperature then fixed and erythrocytes were lysed (Fix/Lyse solution; Thermo Fisher Scientific, UK). Activated GPIIb/IIIa and P-selectin were quantified as median fluorescence intensity of CD41^+^ events. Platelet/leukocyte adhesion was quantified by gating total leukocytes based on scatter characteristics and expressing the percentage of these which were CD41^+^. Thrombosis in vivo was measured using a FeCl_3_ carotid artery injury model as we have done before (*5*). Briefly, under isoflurane anesthesia, the left carotid artery was isolated and 4% ferric chloride (Sigma-Aldrich, UK) was applied to the adventitial surface. Blood flow was measured distally for 30 min using a Doppler flow probe (as above), and the time take for >50% reduction in flow was recorded.

### Statistics and data analysis

Data are presented as means ± SE for *n* independent experiments/animals. Wherever reasonably possible, data were collected and analyzed by an experimenter blinded to the condition under test. Statistical analysis was performed using Prism 8 software (GraphPad Software, USA) using the tests indicated in individual figure legend, and differences were considered significant where *P* < 0.05.

## Supplementary Material

http://advances.sciencemag.org/cgi/content/full/7/12/eabf6054/DC1

Adobe PDF - abf6054_SM.pdf

Endothelial cyclooxygenase-1 paradoxically drives local vasoconstriction and atherogenesis despite underpinning prostacyclin generation

## SUPPLEMENTARY MATERIALS

Supplementary material for this article is available at http://advances.sciencemag.org/cgi/content/full/7/12/eabf6054/DC1

## Appendix

View/request a protocol for this paper from *Bio-protocol*.
