# Supplementary material for: Endothelial cyclooxygenase-1 paradoxically drives local vasoconstriction and atherogenesis despite underpinning prostacyclin generation
Source: Sci Adv. 2021 Mar 19;7(12):eabf6054. doi: 10.1126/sciadv.abf6054 (PMC7978428; doi:10.1126/sciadv.abf6054)
Supplement: http://advances.sciencemag.org/cgi/content/full/7/12/eabf6054/DC1 [file supp_7_12_eabf6054__7.12.eabf6054.DC1.html]

Science Advances | Science AdvancesAAASSearchScience AdvancesMenu

## Supplementary Materials

# Endothelial cyclooxygenase-1 paradoxically drives local vasoconstriction and atherogenesis despite underpinning prostacyclin generation

Jane A. Mitchell, Fisnik Shala, Maria Elisa Lopes Pires, Rachel Y. Loy, Andrew Ravendren, Joshua Benson, Paula Urquhart, Anna Nicolaou, Harvey R. Herschman, Nicholas S. Kirkby

Download Supplement

**This PDF file includes:**

- Figs. S1 and S2
- Table S1

**Files in this Data Supplement:**

- Adobe PDF - abf6054\_SM.pdf
