# Supplementary material for: Endothelial cyclooxygenase-1 paradoxically drives local vasoconstriction and atherogenesis despite underpinning prostacyclin generation
Source: Sci Adv. 2021 Mar 19;7(12):eabf6054. doi: 10.1126/sciadv.abf6054 (PMC7978428; doi:10.1126/sciadv.abf6054)
Supplement: http://advances.sciencemag.org/cgi/content/full/7/12/eabf6054/DC1 [file supp_7_12_eabf6054__abf6054_SM.pdf]

Science Advances
AAAS

advances.sciencemag.org/cgi/content/full/7/12/eabf6054/DC1

Supplementary Materials for

Endothelial cyclooxygenase-1 paradoxically drives local vasoconstriction and atherogenesis despite underpinning prostacyclin generation

Jane A. Mitchell, Fisnik Shala, Maria Elisa Lopes Pires, Rachel Y. Loy, Andrew Ravendren, Joshua Benson, Paula Urquhart, Anna Nicolaou, Harvey R. Herschman, Nicholas S. Kirkby*

*Corresponding author. Email: n.kirkby@imperial.ac.uk

Published 19 March 2021, *Sci. Adv.* **7**, eabf6054 (2021)
DOI: 10.1126/sciadv.abf6054

This PDF file includes:

Figs. S1 and S2
Table S1

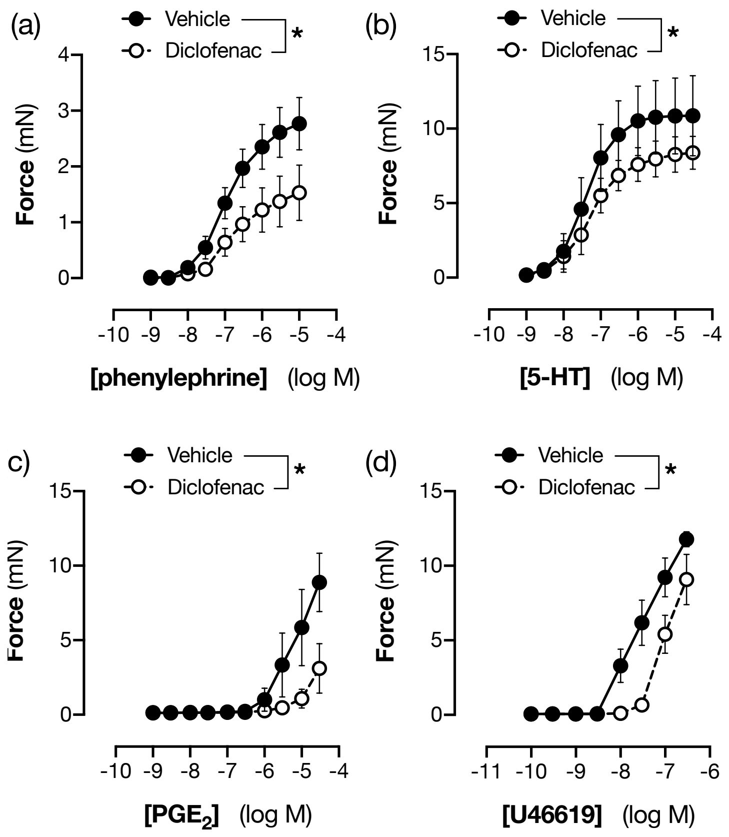

Figure S1: Effect of pharmacological cyclo-oxygenase inhibition on contractile response in mouse aorta. Vasomotor responses to phenylephrine (a; n=8-12), 5-HT (b; n=4), PGE_2 (c; n=4) and the thromboxane mimetic U46619 (d; n=7) in mouse aorta in the presence and absence of the pharmacological cyclo-oxygenase-1/2 inhibitor, diclofenac (1µM). Data are mean ± SEM. *, $p<0.05$ by repeated measures two-way ANOVA.

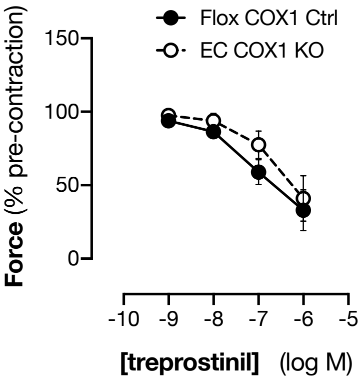

Figure S2: Effect of endothelial cyclo-oxygenase-1 deletion on dilator responses to treprostinil in the mesenteric artery. Vasomotor responses to the prostacyclin analogue treprostinil in phenylephrine pre-contracted mesenteric arteries from control (Flox COX1 Ctrl) and endothelial cyclo-oxygenase-1 knockout mice (EC COX1 KO). Data are mean ± SEM. n=4-5. $p>0.05$ by repeated measures two-way ANOVA.

	Control	Atherosclerosis
**COX-2** (*Ptgs2*)	1.0 ± 0.5	13.7 ± 7.2 *
**COX-1** (*Ptgs1*)	12.5 ± 5.6	23.7 ± 6.8
**mPGES-1** (*Ptges*)	26.0 ± 7.6	47.4 ± 13.7

Table S1: Expression of prostaglandin synthetic enzymes in aortic arches from healthy and atherosclerotic mice. Cyclo-oxygenase-2 (COX-2), cyclo-oxygeanse-1 (COX-1) and microsomal PGE synthase-1 (mPGES-1) expression measured by RT-qPCR in aortic arches from healthy (Flox COX1 Ctrl on normal chow diet) and atherosclerotic (ApoE-deficient Flox COX1 Ctrl mice fed high fat/cholesterol diet for 12 weeks). Expression is normalised (1.0) to levels of COX-2 in the aortic arch of healthy animals. Data are mean ± SEM. n=4. *, $p<0.05$ by Mann-Whitney U-test.
